# Imaging Phenotype of Occupational Endotoxin-Related Lung Function Decline

**DOI:** 10.1289/EHP195

**Published:** 2016-05-03

**Authors:** Peggy S. Lai, Jing-qing Hang, Feng-ying Zhang, J. Sun, Bu-Yong Zheng, Li Su, George R. Washko, David C. Christiani

**Affiliations:** 1 Division of Pulmonary and Critical Care, Massachusetts General Hospital, Boston, Massachusetts, USA; 2 Department of Environmental Health, Harvard T.H. Chan School of Public Health, Boston, Massachusetts, USA; 3 Harvard Medical School, Boston, Massachusetts, USA; 4 Shanghai Putuo District People’s Hospital, Shanghai, China; 5 Division of Pulmonary and Critical Care Medicine, Brigham and Women’s Hospital, Boston, Massachusetts, USA

## Abstract

**Background:**

Although occupational exposures contribute to a significant proportion of obstructive lung disease, the phenotype of obstructive lung disease associated with work-related organic dust exposure independent of smoking remains poorly defined.

**Objective:**

We identified the relative contributions of smoking and occupational endotoxin exposure to parenchymal and airway remodeling as defined by quantitative computed tomography (CT).

**Methods:**

The Shanghai Textile Worker Study is a longitudinal study of endotoxin-exposed cotton workers and endotoxin-unexposed silk workers that was initiated in 1981. Spirometry, occupational endotoxin exposure, and smoking habits were assessed at 5-year intervals. High-resolution computed tomography (CT) was performed in 464 retired workers in 2011, along with quantitative lung densitometric and airway analysis.

**Results:**

Significant differences in all CT measures were noted across exposure groups. Occupational endotoxin exposure was associated with a decrease (–1.3%) in percent emphysema (LAAI-950), a 3.3-Hounsfield unit increase in 15th percentile density, an 18.1-g increase in lung mass, and a 2.3% increase in wall area percent. Current but not former smoking was associated with a similar CT phenotype. Changes in LAAI-950 were highly correlated with 15th percentile density (correlation –1.0). Lung mass was the only measure associated with forced expiratory volume in 1 sec (FEV1) decline, with each 10-g increase in lung mass associated with an additional loss (–6.1 mL) of FEV1 (p = 0.001) between 1981 and 2011.

**Conclusions:**

There are many similarities between the effects of occupational endotoxin exposure and those of tobacco smoke exposure on lung parenchyma and airway remodeling. The effects of occupational endotoxin exposure appear to persist even after the cessation of exposure. LAAI-950 may not be a reliable indicator of emphysema in subjects without spirometric impairment. Lung mass is a CT-based biomarker of accelerated lung function decline.

**Citation:**

Lai PS, Hang J, Zhang F, Sun J, Zheng BY, Su L, Washko GR, Christiani DC. 2016. Imaging phenotype of occupational endotoxin-related lung function decline. Environ Health Perspect 124:1436–1442; http://dx.doi.org/10.1289/EHP195

## Introduction

Chronic obstructive pulmonary disease (COPD) is the fifth leading cause of death in developed countries [Global Burden of Disease (GBD) 2013 Mortality and Causes of Death Collaborators 2015]. Recent population-based studies reported a population-attributable risk of occupational exposures to COPD of 24% overall and 51% among nonsmokers (Mehta et al. 2012). Much of the research characterizing the phenotype and severity of COPD has been performed in smokers (Marchetti et al. 2014; Paulin et al. 2015), where an additional limitation is that all occupational exposures are grouped together under the category of “vapors, gases, dusts, and fumes.” It is well established that different occupational exposures lead to different health effects (Rom and Markowitz 2006), with only some associated with a risk of COPD (Matheson et al. 2005).

Exposure to biological dust is associated with an increased risk of COPD (Matheson et al. 2005). Furthermore, exposure to endotoxin in organic dust is common, with high-level exposures measured in both work environments [farms, cotton processing facilities, and animal care facilities (Liebers et al. 2008)] and nonwork environments [schools (Jacobs et al. 2013) and homes that burn biomass fuel (Semple et al. 2010)], highlighting the public health relevance of this exposure. Repeated inhalation of endotoxin at levels approximating those found in occupational settings has led to the development of both emphysema (Brass et al. 2008) and airways disease (Brass et al. 2003) in murine models. Therefore, the study of lung disease in the setting of occupational endotoxin exposure represents an opportunity to further characterize the phenotype of COPD associated with an important environmental exposure.

The Shanghai Textile Worker Study is an occupational cohort that has been followed longitudinally since 1981. Unique to this cohort are exposure characterization over the entire working lifetime of the participants, a large proportion of nonsmokers, and little loss to follow-up, with 74% of the original participants still alive participating in the 30-year survey. For decades, controversy has existed surrounding whether emphysema or airways disease forms the basis for the chronic airflow obstruction noted in the setting of endotoxin-containing cotton dust exposure. Prior autopsy studies have demonstrated both, although these studies could not distinguish between disease caused by concurrent smoking and disease caused by work exposure (Edwards et al. 1975; Pratt et al. 1980; Schachter et al. 1980). In this study, our primary aim was to identify the relative contributions of smoking and occupational endotoxin exposure to parenchymal and airway remodeling as defined by quantitative computed tomography (CT). Our secondary aim was to identify imaging biomarkers associated with lung function decline.

## Methods

### Study Population and Study Design

**Figure 1:**
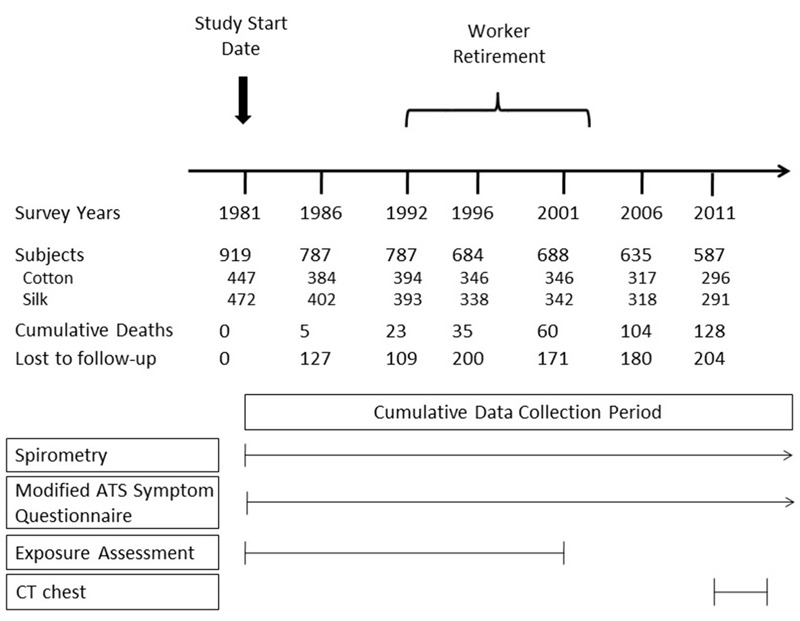
Overview of Shanghai Textile Worker Study. Most of the workers retired between 1992 and 2001. Of the 587 subjects in the 2011 follow-up survey, 464 consented to and received high-resolution computed tomography of the chest. Abbreviations: ATS, American Thoracic Society; CT, computed tomography.

The Shanghai Textile Worker Study was designed based on the observation that cotton dust contains high levels of endotoxin, whereas silk dust contains undetectable levels of endotoxin, representing a natural experiment to evaluate the effects of occupational endotoxin exposure on lung disease. In 1981, 919 Han Chinese workers were recruited from two cotton mills and one silk textile mill in the same industrial sector of Shanghai, China (study schema in Figure 1); 90% of those eligible were enrolled (Christiani et al. 1994). The main inclusion criterion was at least 2 years of work in the identified mills in order to ensure a stable study population; subjects with a history of prior respiratory disease were excluded. Cotton and silk workers were comparable with respect to income, place of residence, and other socio-economic factors owing to the hiring practices of the Shanghai Textile Bureau. Surveys were performed in 1981, 1986, 1992, 1996, 2001, 2006, and 2011, with eligibility for retesting based on presence in the baseline 1981 survey. Prebronchodilator spirometry performed according to American Thoracic Society guidelines; a physical exam; modified American Thoracic Society symptom, work history, and smoking questionnaires; and exposure assessment (in the period before worker retirement) were performed at each survey.

In 2011, 464 subjects consented to volumetric chest CT scans performed at full inspiration and expiration using a single Siemens Emotion-16 CT scanner. Images were obtained at 0.75-mm slice thickness and were reconstructed using a B65s reconstruction kernel. Airway Inspector software (San Jose Estepar et al. 2008) was used to obtain measures of both lung attenuation and airway morphology. In addition, all scans were interpreted based on a consensus read by two radiologists reading simultaneously onto a standardized CT assessment score sheet to identify the presence of emphysema.

Written informed consent was obtained from all subjects, and the study was approved by the Institutional Review Boards at the Harvard T.H. Chan School of Public Health and the Shanghai Putuo District People’s Hospital.

### Exposure Assessment

Exposure assessment was performed as previously described (Kennedy et al. 1987; Olenchock et al. 1990). Between 1981 and 2001 (after which most workers retired), multiple area samples were collected from each of the six different work areas in the two cotton mills and the one silk mill. Vertical elutriators were used to collect respirable fractions of dust, with sampling times ranging from 3 to 7 hr. All collected dust samples were weighed to estimate exposure to respirable dust, and dust from all cotton mills and a limited number of full-shift samples from silk mills obtained during that period were sent to a single laboratory at the National Institute of Occupational Safety and Health to quantify endotoxin content using a Limulus amebocyte lysate gel test (Pyrostat-50; Worthington Biochemical Corporation). Values for each filter were summed and converted from nanograms/milliliter to micrograms/cubic meter based on sampling time and air flow rates of each sampler. The lower limit of detection for endotoxin by this method was 0.001 endotoxin units (EU)/m^3^. Exposure measurements collected in the first survey were used to estimate pre-1981 exposure by area, and area exposure measurements in 2001 were used to approximate post-2001 area exposures. Six full-shift samples from the silk mill confirmed near-undetectable levels of endotoxin (0.001 EU/m^3^) in the vertical elutriator samples; thus, silk workers were considered to be exposed to negligible amounts of endotoxin at work. Individual endotoxin exposure was calculated using geometric means of endotoxin measured in each work area multiplied by years of work in each work area, resulting in a lifetime cumulative index of occupational exposure measured in endotoxin units/cubic meter-years (EU/m^3^-years), with an interpretation analogous to that of pack-years for smoking. At each survey, a detailed work history was obtained to identify the date of textile work cessation; post-retirement job descriptions were also obtained to account for occupational endotoxin exposure after the workers had officially retired from the mills.

### Outcome Measures

The primary outcome measures were CT measures of parenchymal remodeling and airway morphology. To evaluate parenchymal remodeling, we evaluated the following measures: *a*) percent emphysema defined by the percentage of low attenuation area of lung < –950 Hounsfield units (HU) at full inspiration (LAA_I-950_); *b*) 15th percentile density, which is the HU threshold demarcating the lowest 15th percent of lung attenuation (PD15) (Parr et al. 2008); and *c*) lung mass (Henne et al. 2012), based on studies suggesting that emphysematous destruction of the lung parenchyma may be associated with increased lung mass caused by inflammation and remodeling (Guenard et al. 1992).

Measures of central airway morphology to identify wall area percent (WA %) were obtained in all subjects from the apical segment of the right upper lobe, a third-generation segmental airway. Airway segmentation was performed using phase congruency as described previously (Estépar et al. 2006).

This investigation utilized a single CT scanner that was calibrated to ensure accuracy. The protocol consisted of daily air calibrations as well as periodic water calibrations according to the vendor’s recommendations. Air and water have known attenuation values of –1,000 HU and 0 HU respectively, with attenuation values for emphysema (Gevenois et al. 1995), normal lung (Coxson et al. 1999), and interstitial abnormalities (Lederer et al. 2009) falling within this range. Validation of the extremes of this range allows accurate densitometric discrimination of processes affecting the lung tissue.

The secondary outcome measure was change in forced expiratory volume in 1 sec (FEV_1_) between 1981 and 2011, calculated as the difference in FEV_1_ measured at these two time points.

### Statistical Analysis

In the primary outcome analysis, linear regression was used to determine the association between exposure and each CT outcome measure. Occupational endotoxin exposure was modeled in one of two ways; either using cotton versus silk work as a binary variable or as log-transformed cumulative occupational endotoxin exposure. Smoking exposure was modeled using smoking status (defined as never, ever, or former) and cumulative pack-years smoked. All multivariable analyses were adjusted for age, sex, height, body mass index, duration of work-cessation years, and inflation level. For inspiratory measures, inflation level was calculated using CT-measured total volume divided by predicted total lung capacity (Grydeland et al. 2010). Sensitivity analyses were performed in which the analysis was restricted to cotton workers (given that only a limited number of full-shift measures for endotoxin was performed in the silk workers), nonsmokers, and cotton workers who were nonsmokers. Additional sensitivity analyses were performed with models incorporating interaction terms between occupational and smoking exposure.

In the secondary outcome measure, CT measures of remodeling were included as additional covariates in order to identify predictors of lung function decline.

All analyses were performed in R v.3.1 (R Core Team 2015). Two-sided *p*-values of < 0.05 were considered significant.

## Results

Characteristics of the 464 subjects are presented in Table 1. Most (70.2%) were lifetime nonsmokers, and 52.6% were cotton workers. All subjects had retired from active textile work, with the average duration of retirement being 17.7 ± 4.6 years. A total of 168 (36.2%) were male, and the average age was 63.6 ± 8.7 years old. Notably, 93% of all smokers were male, whereas 89% of all nonsmokers were female. Unlike smoking exposure, occupational exposure was not stratified by sex; 35% of silk workers and 37.2% of cotton workers were male. The average percent predicted FEV_1_ was 109.7 ± 17.8%; 7 nonsmokers and 16 smokers had FEV_1_/FVC below the lower limit of normal. Annual declines in FEV_1_ were –15.1, –18.3, –28.4, and –31.9 mL/year for nonsmoking silk workers, nonsmoking cotton workers, smoking silk workers, and smoking cotton workers, respectively (*p* = 0.008 for linear trend).

**Table 1:** Baseline characteristics of study participants stratified by exposure level in 2011.

Characteristic	Silk nonsmoker	Cotton nonsmoker	Silk smoker	Cotton smoker
Observations, *n*	158	164	62	80
Age, years	63.5 ± 8.8	63.8 ± 8.7	65.8 ± 9.9	63.8 ± 9.3
Male	17 (10.8%)	19 (11.6%)	60 (96.8%)	72 (90.0%)
Current smoker	0 (0%)	0 (0%)	43 (69.4%)	58 (72.5%)
Pack-years	0	0	28.4 ± 20.5	27.5 ± 18.6
Height, cm	157.9 ± 6	159.2 ± 6.5	167.2 ± 6.1	168.2 ± 7.2
Body mass index, cm/kg^2^	23.8 ± 3.0	24.9 ± 3.6	24.0 ± 3.2	24.6 ± 3.2
Follow-up time, years	29.4 ± 0.1	29.6 ± 0.1	29.3 ± 0.0	29.6 ± 0.0
Work duration, years	25.4 ± 8.2	24.5 ± 7.3	28.5 ± 9.7	26.6 ± 7.9
Retirement duration, years	18.4 ± 4.1	17.4 ± 5.1	17.4 ± 4.3	16.9 ± 4.7
Cumulative endotoxin exposure, EU/m^3^-years^*a*^	0	38233.8 ± 31962.6	0	61123.6 ± 54849.7
FEV_1_, mL	2159.8 ± 440.4	2193.0 ± 491.5	2683.3 ± 678.0	2540.3 ± 811.7
FEV_1_, % predicted	112.0 ± 16.8	111.4 ± 14.7	110.4 ± 21.3	100.4 ± 20.5
FEV_1_/FVC	0.8 ± 0.1	0.8 ± 0.1	0.7 ± 0.1	0.7 ± 0.1
FEV_1_/FVC < 0.7	15 (9.5%)	14 (8.5%)	19 (30.6%)	19 (23.8%)
FEV_1_/FVC < LLN	4 (2.5%)	3 (1.8%)	9 (14.5%)	7 (8.8%)
Annual FEV_1_ decline, mL/yr^*b*^	–15.1 ± 10.4	–18.3 ± 9.4	–28.4 ± 11.8	–31.9 ± 13.6
FEV_1_ change since 1981, mL^*c*^	–442.0 ± 304.2	–542.3 ± 179.5	–832.8 ± 347.3	–941.9 ± 401.2
Any respiratory symptoms	38 (24.1%)	38 (23.2%)	22 (35.5%)	29 (36.2%)
Chronic bronchitis	6 (3.8%)	8 (4.9%)	9 (14.5%)	14 (17.5%)
Chronic cough	4 (2.5%)	3 (1.8%)	3 (4.8%)	3 (3.8%)
Dyspnea	35 (22.2%)	36 (22.0%)	14 (22.6%)	21 (26.2%)
Abbreviations: EU, endotoxin units; FEV_1_, forced expiratory volume in 1 sec; FVC, forced vital capacity; LLN, lower limit of normal. ^***a***^A limited number of full-shift samples obtained in silk mills had endotoxin levels below the lower limit of normal (0.001 EU/m^3^) by the Limulus amoebocyte lysate assay; thus, silk workers were considered to be exposed to negligible amounts of endotoxin at work. ^***b***^Calculated by taking the difference in FEV_1_ between 2011 and 1981 divided by elapsed time. Significant differences in annual FEV_1_ decline noted between exposure groups; *p* = 0.008 for linear trend. ^***c***^Significant differences in FEV_1_ between 1981 and 2011; *p* < 0.001 for linear trend.				

Quantitative CT characteristics of the study population are given in Table 2. There were significant differences in CT measures across all exposure groups. In these unadjusted analyses, cotton work was associated with lower percent emphysema, higher 15th percentile density, greater lung mass, and higher wall area percent, whereas smoking was associated with higher percent emphysema, lower 15th percentile density, greater lung mass, and lower wall area percent. It must be emphasized that most smokers were male and most nonsmokers were female; large sex differences in quantitative CT measures have previously been described, with men having higher percent emphysema than women (Grydeland et al. 2009; Hoffman et al. 2014).

**Table 2:** Quantitative computed tomography characteristics of study population.

Characteristic	Silk nonsmoker	Cotton nonsmoker	Silk smoker	Cotton smoker	*p*-Value^*a*^
% Emphysema (LAA_I-950_)	12.5 ± 6.2	11.9 ± 5.6	16.3 ± 6.2	14.0 ± 6.2	< 0.001
15th Percentile density (PD15)	–938.3 ± 26.5	–936.8 ± 27.0	–950.1 ± 22.6	–942.8 ± 25.7	0.004
Lung mass, g	616.5 ± 80.4	653.7 ± 91.4	775.7 ± 120.7	799.4 ± 123.5	< 0.001
Wall area %	59.9 ± 6.4	62.7 ± 6.5	58.7 ± 6.2	60.0 ± 6.2	< 0.001
^***a***^Based on one-way analysis of variance.					

The associations between occupational or smoking exposure and quantitative CT measures in multivariate models are shown in Table 3. In the overall cohort, cotton work was associated with significant decreases in percent emphysema, increases in 15th percentile density, lung mass, and wall area percent. When occupational exposure was modeled as cumulative endotoxin exposure, a dose–response relationship was observed. Increased endotoxin exposure was associated with decreased percent emphysema, increased 15th percentile density, increased lung mass, and increased wall area percent.

**Table 3:** Multivariate mean differences in computed tomography measures of lung parenchyma and airway remodeling based on exposure.*^a^* Results based on all participants (*n* = 464) and restricted to cotton workers (*n* = 244), nonsmokers (*n* = 322), and cotton workers who were nonsmokers (*n* = 164). Interaction terms between occupational exposure and smoking were non-significant.

Exposure	% Emphysema (LAA_I-950_)	15th Percentile density (PD15)	Lung mass	Wall area %
All
Cotton vs. silk	–1.26*** [–2.06, –0.46]	3.30** [0.12, 6.48]	18.10*** [4.52, 31.68]	2.32*** [1.17, 3.48]
Cumulative endotoxin, log(EU/m^3^)	–0.05*** [–0.07, –0.02]	0.12** [0.01, 0.24]	0.67*** [0.20, 1.15]	0.08*** [0.04, 0.12]
Current vs. never smoker	–2.39** [–4.20, –0.58]	10.11*** [2.91, 17.30]	47.38*** [16.66, 78.10]	1.87 [–0.71, 4.46]
Former vs. never smoker	0.44 [–1.36, 2.25]	0.14 [–7.02, 7.30]	–0.56 [–31.13, 30.02]	0.27 [–2.32, 2.87]
Pack-years	0.01 [–0.03, 0.04]	–0.07 [–0.22, 0.08]	0.23 [–0.41, 0.87]	–0.03 [–0.08, 0.03]
Cotton
Cumulative endotoxin, log(EU/m^3^)	–0.30* [–0.65, 0.04]	1.33* [–0.06, 2.73]	6.77** [0.91, 12.63]	–0.36 [–0.88, 0.16]
Current vs. never smoker	–1.37 [–3.78, 1.03]	8.89* [–0.84, 18.63]	33.61 [–7.37, 74.58]	–0.35 [–3.94, 3.24]
Former vs. never smoker	0.08 [–2.32, 2.47]	2.45 [–7.24, 12.13]	4.19 [–36.57, 44.95]	0.15 [–3.45, 3.75]
Pack-years	–0.001 [–0.06, 0.05]	–0.08 [–0.30, 0.14]	–0.07 [–1.00, 0.85]	0.03 [–0.05, 0.11]
Non-smokers
Cotton vs. silk work	–1.26*** [–2.06, –0.46]	3.30* [–0.57, 7.17]	21.18*** [5.63, 36.73]	2.94*** [1.56, 4.32]
Cumulative endotoxin, log(EU/m^3^)	–0.05*** [–0.07, –0.02]	0.13* [–0.005, 0.27]	0.82*** [0.27, 1.37]	0.10*** [0.05, 0.15]
Cotton non-smokers
Cumulative endotoxin, log(EU/m^3^)	–0.75*** [–1.19, –0.31]	2.79*** [0.90, 4.69]	12.89*** [5.28, 20.49]	–0.003 [–0.68, 0.67]
EU, endotoxin units. ^***a***^All models were adjusted for age, sex, height, body mass index, duration of work-cessation years, and inflation or deflation level using computed tomography (CT)-measured volumes divided by predicted volumes. *, *p* < 0.1; **, *p* < 0.05; ***, *p* < 0.01.				

When the analysis was restricted to cotton workers (*n* = 244), an association between occupational endotoxin exposure and lung mass was still detected, with a trend towards significance in the associations between occupational exposure and percent emphysema and 15th percentile density. When the analysis was restricted to cotton workers who were nonsmokers (*n* = 164), significant associations between occupational endotoxin exposure and decreased percent emphysema, increased 15th percentile density, and increased lung mass were detected. When the analysis was restricted to nonsmokers (*n* = 322), highly similar effect estimates were observed. None of the interaction terms between occupational and smoking exposure was significant for any of the outcomes.

In the overall cohort, the adjusted effect of smoking on quantitative CT measures was very similar in direction to that of occupational endotoxin exposure. Compared with never smokers, current smokers had lower percent emphysema, higher 15th percentile density, greater lung mass, and nonsignificant increases in wall area percent. However, this association was not seen when comparing former versus never smokers or when evaluating pack-years.

**Figure 2:**
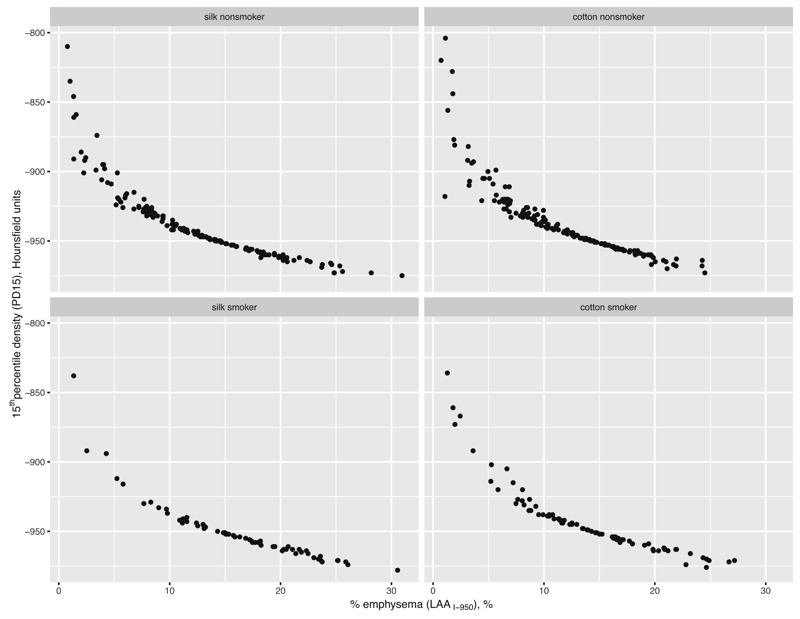
Correlation between CT measure of percent emphysema (using cutoff of –950 Hounsfield units, LAA_I-950_) and 15th percentile density across all exposure subgroups. Correlation was high (Pearson correlation –0.87, Spearman correlation –1.00) and suggested that the percent emphysema measure may actually be reflecting changes in lung density, and not a true measure of emphysema.

To better understand the unexpected association between occupational or smoking exposures and decreased percent emphysema as measured by LAA_I-950_, additional analyses were performed. Measures of LAA_I-950_ were highly correlated with 15th percentile density (Pearson correlation –0.87, Spearman correlation –1.00), with lower measures of percent emphysema associated with higher 15th percentile density in all exposure groups (Figure 2). Additionally, all CT scans were reviewed by two radiologists for the presence or absence of emphysema to compare the distribution of LAA_I-950_ in participants with versus those without emphysema. Average LAA_I-950_ was 13.9% in participants with emphysyma versus 12.7% in those without emphysema (*p* = 0.06). Notably, LAA_I-950_ ranged from 0.7% to 30.6% versus 0.8% to 31.0% in participants with emphysema versus those without emphysema, indicating significant overlap.

In multivariable analyses, lung mass was the only CT measure that was significantly associated with lung function decline from 1981 to 2011 (Table 4). Each 10-g increase in lung mass was associated with an additional loss (–6.1 mL) of FEV_1_ (*p* = 0.001) over this 30-year period.

**Table 4:** Quantitative CT measures associated with decline in FEV_1_ between 1981 and 2011.

Characteristic	FEV_1_ change (mL)^*a*^
% emphysema (LAA_I-950_)	1.26 [–3.53, 6.04]
15th percentile density, Hounsfield units	–0.37 [–1.46, 0.72]
Lung mass, g	–0.61*** [–0.97, –0.24]
Wall area %	0.48 [–4.14, 5.10]
Abbreviations: CT, computed tomography; FEV_1_, forced expiratory volume in 1 sec. Lung mass was the only CT measure associated with lung function decline. Each 10-g increase in lung mass was associated with an additional loss (–6.1 mL) of FEV_1_ over the 30-year study period. All models were adjusted for age, sex, height, body mass index, duration of work-cessation years, occupational exposure (cotton vs. silk work), and smoking exposure (never, ever, former smoker as well as pack-years smoked). ^***a***^FEV_1_ change associated with 1-g increase in lung mass. ***, p < 0.01.	

## Discussion

This is the first comprehensive occupational study evaluating the relative contributions of lifetime occupational endotoxin and smoking exposure to the phenotype of lung function decline as defined by quantitative lung CT. In our cohort, we found that workplace exposures led to a phenotype of lung disease very similar to that of smoking exposure, with increased lung density and increased lung mass. Occupational endotoxin exposure was also associated with increased airway wall thickening in the overall cohort. LAA_I-950_ did not appear to be a reliable measure of emphysema when compared with radiologist review in our cohort and may have reflected changes in overall lung density rather than the presence of emphysema. Lung mass was the only CT biomarker associated with longitudinal FEV_1_ decline.

Our study is the only available study that quantifies the degree to which lifetime occupational endotoxin exposure in cotton textile workers contributes to the phenotype of lung disease as defined by quantitative lung CT. There is only one other study that has investigated the CT phenotype of lung disease associated with occupational exposures (Marchetti et al. 2014); this report was based on the COPDGene study, where participants were all current or former smokers, and 86% met spirometric criteria for COPD. Self-reported ever exposure to dust or fumes was associated with increased percent emphysema, in contrast to our findings. However, only 10.8% of our cohort met spirometric criteria for COPD owing to the healthy-worker survivor effect common in many occupational studies.

An apparent “paradoxical” increase in CT measures of percent emphysema has been noted when smokers quit (Shaker et al. 2011). In a study of smokers with COPD followed with annual CT scans, both quitting smoking and budesonide use were associated with an increase in percent emphysema and a decrease in PD15. These activities are presumably antiinflammatory and should not worsen emphysema. Percent emphysema has an almost perfect inverse correlation with PD15, and the decrease in percent emphysema associated with both smoking and occupational exposures in our study likely reflects increased lung density caused by inflammation rather than an actual decrease in emphysema associated with these noxious environmental exposures. Another population-based study found lower percent emphysema (defined by LAA_I-950_) in current compared with former smokers, although the authors speculated that this finding was the result of a healthy-smoker survivor effect (Grydeland et al. 2009). The use of LAA_I-950_ to quantify the extent of emphysema was originally validated in cohorts of subjects with spirometric COPD; it is unclear, as we have found, whether this sensitive measure is well correlated with the extent of emphysema in healthier subjects as seen in population-based or occupational cohorts.

We found many phenotypic similarities between the effects of active smoking and those of occupational endotoxin exposure on parenchyma and airway changes. It is interesting that whereas only active (and not former) smoking was associated with changes in lung density, prior occupational endotoxin exposure was associated with persistent changes in lung density and airway wall thickening. At the time imaging was performed, the average duration of retirement was 17.7 years in this cohort. The effects of occupational endotoxin exposure on lung morphology appear to have persisted even after cessation of exposure. We have previously shown that in this cohort, prior occupational endotoxin exposure was associated with a dose-related impairment in lung function recovery even after cessation of exposure (Lai et al. 2015); this finding suggests that these persistent CT changes have functional significance.

The mechanism of these persistent CT changes despite exposure cessation is not clear because few animal and human studies have evaluated chronic rather than acute endotoxin exposure. We do not think that subclinical interstitial lung disease caused by occupational endotoxin exposure can explain the persistent increase in lung density and lung mass noted here because 32 (6.9%) silk workers and 31 (6.7%) cotton workers had interstitial lung abnormalities based on a validated radiologist sequential reading method (Washko et al. 2010). A murine model of chronic endotoxin exposure showed persistent lung neutrophilic inflammation that was correlated with an expansion of lung inflammatory dendritic cells (Lai et al. 2012), suggesting that if these persistent changes in lung density are caused by persistent inflammation despite exposure cessation, then a plausible mechanism to explain these findings does exist.

We identified lung mass as the only CT-based biomarker associated with FEV_1_ decline. Although other studies have supported the association between accelerated FEV_1_ decline and emphysema extent as measured by LAA_I-950_ (Vestbo et al. 2011) or by radiologist assessment (Nishimura et al. 2012), these studies were performed in subjects with a spirometric diagnosis of COPD, and the results may not be applicable to population-based or occupational cohorts without evidence of lung function impairment. Adjusting for age, sex, and anthropometric measures, each 10-g increase in lung mass was associated with an additional 6.1-mL loss in FEV_1_ over the study period. Lung mass measured by CT has been validated against *ex vivo* measurements (Henne et al. 2012), and studies have revealed the unexpected finding that patients with emphysema appear to have heavier rather than lighter lungs (Guenard et al. 1992). In *ex vivo* lungs from patients with emphysema, airspace enlargement was accompanied by an even greater increase in both elastin and collagen in the alveolar interstitium (Vlahovic et al. 1999). This finding provides a plausible mechanistic explanation for why lung mass was the most potent marker for disease activity as measured by lung function decline in our cohort; after adjusting for age, sex, and anthropometric differences, lung mass may be the optimal biomarker for inflammation or for parenchymal remodeling.

Our study has several strengths. First, to our knowledge, this is the only study that has estimated measured workplace endotoxin exposures over the working lifetime of a cohort of cotton textile workers. Other occupational studies of lung disease have relied on self-reported exposures, which are influenced by recall bias, or job exposure matrices, which cannot quantify actual exposures. Second, our cohort has a large proportion of nonsmokers, whereas most other studies evaluating the contribution of occupational exposures to lung disease were performed largely in smokers. Third, follow-up in our cohort has spanned three decades, allowing us to obtain estimates of FEV_1_ decline over this period of time. Although cross-sectional measures of percent predicted FEV_1_ were within the normal range in this cohort, there were differences in FEV_1_ decline when comparing exposure groups, highlighting the importance of longitudinal studies when assessing the impact of exposure on respiratory outcomes. Our findings suggest that in the absence of longitudinal lung function data, cross-sectional CT measures of lung mass may serve as a biomarker for accelerated lung function decline.

Our study also has several limitations. First, smoking and sex were largely confounded because most smokers were male, and most nonsmokers were female. However, an analysis restricted to nonsmokers and cotton workers who were nonsmokers showed very similar results to those from our overall cohort, supporting our conclusions. Second, cotton dust may contain other microbial (Rylander et al. 1985) or bioactive (Buck et al. 1986) compounds for which endotoxin may serve as a proxy. We did not measure bioactive agents in cotton dust other than endotoxin because the association between endotoxin in cotton dust and health effects has been the most robustly supported association in the literature (Lai and Christiani 2013). Third, because of the healthy-worker survivor effect seen in many occupational cohorts, only 10.8% of our cohort met spirometric criteria for COPD, and longitudinal FEV_1_ changes were small compared with population controls. However, an important research initiative is the detection of subclinical disease using noninvasive imaging methods to identify individuals at a stage where interventions can prevent disease development or progression. Therefore, our finding of lung mass as an important biomarker for FEV_1_ decline in both smoking and occupational organic dust exposure suggests that it can be more broadly applied to other COPD-related exposures.

## Conclusions

In conclusion, in this workplace-based study of occupational organic dust and smoking exposures, we found that occupational endotoxin exposure was associated with persistent increases in lung density, lung mass, and airway thickening even after exposure cessation. CT measures of percent emphysema using LAA_I-950_ are likely more reflective of lung density rather than of emphysema in populations without overt clinical disease and should be interpreted with caution. Lung mass represents a potential CT-based biomarker for lung function decline and should be further validated in future studies.

## References

[r1] Brass DM, Hollingsworth JW, Cinque M, Li Z, Potts E, Toloza E (2008). Chronic LPS inhalation causes emphysema-like changes in mouse lung that are associated with apoptosis.. Am J Respir Cell Mol Biol.

[r2] Brass DM, Savov JD, Gavett SH, Haykal-Coates N, Schwartz DA (2003). Subchronic endotoxin inhalation causes persistent airway disease.. Am J Physiol Lung Cell Mol Physiol.

[r3] Buck MG, Wall JH, Schachter EN (1986). Airway constrictor response to cotton bract extracts in the absence of endotoxin.. Br J Ind Med.

[r4] Christiani DC, Ye TT, Wegman DH, Eisen EA, Dai HL, Lu PL (1994). Cotton dust exposure, across-shift drop in FEV1, and five-year change in lung function.. Am J Respir Crit Care Med.

[r5] Coxson HO, Rogers RM, Whittall KP, D’Yachkova Y, Paré PD, Sciurba FC (1999). A quantification of the lung surface area in emphysema using computed tomography.. Am J Respir Crit Care Med.

[r6] Edwards C, Macartney J, Rooke G, Ward F (1975). The pathology of the lung in byssinotics.. Thorax.

[r7] Estépar RS, Washko GG, Silverman EK, Reilly JJ, Kikinis R, Westin CF (2006). Accurate airway wall estimation using phase congruency.. Med Image Comput Comput Assist Interv.

[r8] GBD 2013 Mortality and Causes of Death Collaborators (2015). Global, regional, and national age-sex specific all-cause and cause-specific mortality for 240 causes of death, 1990–2013: a systematic analysis for the Global Burden of Disease Study 2013..

[r9] Gevenois PA, de Maertelaer V, De Vuyst P, Zanen J, Yernault JC (1995). Comparison of computed density and macroscopic morphometry in pulmonary emphysema.. Am J Respir Crit Care Med.

[r10] Grydeland TB, Dirksen A, Coxson HO, Eagan TM, Thorsen E, Pillai SG (2010). Quantitative computed tomography measures of emphysema and airway wall thickness are related to respiratory symptoms.. Am J Respir Crit Care Med.

[r11] Grydeland TB, Dirksen A, Coxson HO, Pillai SG, Sharma S, Eide GE (2009). Quantitative computed tomography: emphysema and airway wall thickness by sex, age and smoking.. Eur Respir J.

[r12] Guenard H, Diallo MH, Laurent F, Vergeret J (1992). Lung density and lung mass in emphysema.. Chest.

[r13] Henne E Anderson JC Lowe N Kesten S 2012 Comparison of human lung tissue mass measurements from *ex vivo* lungs and high resolution CT software analysis. BMC Pulm Med 12 18, doi:10.1186/1471-2466-12-18 22584018 PMC3499450

[r14] Hoffman EA, Ahmed FS, Baumhauer H, Budoff M, Carr JJ, Kronmal R (2014). Variation in the percent of emphysema-like lung in a healthy, nonsmoking multiethnic sample. The MESA Lung Study.. Ann Am Thorac Soc.

[r15] Jacobs JH, Krop EJ, de Wind S, Spithoven J, Heederik DJ (2013). Endotoxin levels in homes and classrooms of Dutch school children and respiratory health.. Eur Respir J.

[r16] Kennedy SM, Christiani DC, Eisen EA, Wegman DH, Greaves IA, Olenchock SA (1987). Cotton dust and endotoxin exposure-response relationships in cotton textile workers.. Am Rev Respir Dis.

[r17] Lai PS, Christiani DC (2013). Long-term respiratory health effects in textile workers.. Curr Opin Pulm Med.

[r18] Lai PS, Fresco JM, Pinilla MA, Macias AA, Brown RD, Englert JA (2012). Chronic endotoxin exposure produces airflow obstruction and lung dendritic cell expansion.. Am J Respir Cell Mol Biol.

[r19] Lai PS, Hang JQ, Valeri L, Zhang FY, Zheng BY, Mehta AJ (2015). Endotoxin and gender modify lung function recovery after occupational organic dust exposure: a 30-year study.. Occup Environ Med.

[r20] Lederer DJ, Enright PL, Kawut SM, Hoffman EA, Hunninghake G, van Beek EJ (2009). Cigarette smoking is associated with subclinical parenchymal lung disease: the Multi-Ethnic Study of Atherosclerosis (MESA)-Lung Study.. Am J Respir Crit Care Med.

[r21] Liebers V Raulf-Heimsoth M Brüning T 2008 Health effects due to endotoxin inhalation (review) Arch Toxicol 82 203 210 18322674 10.1007/s00204-008-0290-1

[r22] Marchetti N, Garshick E, Kinney GL, McKenzie A, Stinson D, Lutz SM (2014). Association between occupational exposure and lung function, respiratory symptoms, and high-resolution computed tomography imaging in COPDGene.. Am J Respir Crit Care Med.

[r23] Matheson MC, Benke G, Raven J, Sim MR, Kromhout H, Vermeulen R (2005). Biological dust exposure in the workplace is a risk factor for chronic obstructive pulmonary disease.. Thorax.

[r24] Mehta AJ, Miedinger D, Keidel D, Bettschart R, Bircher A, Bridevaux PO (2012). Occupational exposure to dusts, gases, and fumes and incidence of chronic obstructive pulmonary disease in the Swiss Cohort Study on Air Pollution and Lung and Heart Diseases in Adults.. Am J Respir Crit Care Med.

[r25] Nishimura M, Makita H, Nagai K, Konno S, Nasuhara Y, Hasegawa M (2012). Annual change in pulmonary function and clinical phenotype in chronic obstructive pulmonary disease.. Am J Respir Crit Care Med.

[r26] Olenchock SA, Christiani DC, Mull JC, Ye TT, Lu PL (1990). Airborne endotoxin concentrations in various work areas within two cotton textile mills in the People’s Republic of China.. Biomed Environ Sci.

[r27] Parr DG Sevenoaks M Deng C Stoel BC Stockley RA 2008 Detection of emphysema progression in alpha 1-antitrypsin deficiency using CT densitometry; methodological advances. Respir Res 9 21, doi:10.1186/1465-9921-9-21 18271964 PMC2287169

[r28] Paulin LM, Diette GB, Blanc PD, Putcha N, Eisner MD, Kanner RE (2015). Occupational exposures are associated with worse morbidity in patients with chronic obstructive pulmonary disease.. Am J Respir Crit Care Med.

[r29] Pratt PC, Vollmer RT, Miller JA (1980). Epidemiology of pulmonary lesions in nontextile and cotton textile workers: a retrospective autopsy analysis.. Arch Environ Health.

[r30] R Core Team 2015 R: A Language and Environment for Statistical Computing. Austria, Vienna R Foundation for Statistical Computing http://www.R-project.org [accessed 28 November 2015].

[r31] Rom WN, Markowitz S, eds (2006). Environmental and Occupational Medicine. 4th ed..

[r32] Rylander R, Haglind P, Lundholm M (1985). Endotoxin in cotton dust and respiratory function decrement among cotton workers in an experimental cardroom.. Am Rev Respir Dis.

[r33] San Jose Estepar R, Washko GG, Silverman EK, Reilly JJ, Kikinis R, Westin CF (2008). Airway inspector: an open source application for lung morphometry.. In: Proceedings of the First International Workshop on Pulmonary Image Processing, 6 September 2008. New York City, New York.

[r34] Schachter EN, Beck GJ, Maunder LR (1980). A “retrospective” analysis of autopsy data concerning pathologic lesions in the lungs of cotton textile workers.. Arch Environ Health.

[r35] Semple S Devakumar D Fullerton DG Thorne PS Metwali N Costello A 2010 Airborne endotoxin concentrations in homes burning biomass fuel. Environ Health Perspect 118 988 991, doi:10.1289/ehp.0901605 20308032 PMC2920920

[r36] Shaker SB, Stavngaard T, Laursen LC, Stoel BC, Dirksen A (2011). Rapid fall in lung density following smoking cessation in COPD.. COPD.

[r37] Vestbo J, Edwards LD, Scanlon PD, Yates JC, Agusti A, Bakke P (2011). Changes in forced expiratory volume in 1 second over time in COPD.. N Engl J Med.

[r38] Vlahovic G, Russell ML, Mercer RR, Crapo JD (1999). Cellular and connective tissue changes in alveolar septal walls in emphysema.. Am J Respir Crit Care Med.

[r39] Washko GR, Lynch DA, Matsuoka S, Ross JC, Umeoka S, Diaz A (2010). Identification of early interstitial lung disease in smokers from the COPDGene Study.. Acad Radiol.

